# Facial Expression Aftereffect Revealed by Adaption to Emotion-Invisible Dynamic Bubbled Faces

**DOI:** 10.1371/journal.pone.0145877

**Published:** 2015-12-30

**Authors:** Chengwen Luo, Qingyun Wang, Philippe G. Schyns, Frederick A. A. Kingdom, Hong Xu

**Affiliations:** 1 Division of Psychology, Nanyang Technological University, Singapore, Singapore; 2 Institute of Neuroscience and Psychology, University of Glasgow, Glasgow, United Kingdom; 3 McGill Vision Research, Department of Ophthalmology, McGill University, Montreal, Canada; Tilburg University, NETHERLANDS

## Abstract

Visual adaptation is a powerful tool to probe the short-term plasticity of the visual system. Adapting to local features such as the oriented lines can distort our judgment of subsequently presented lines, the tilt aftereffect. The tilt aftereffect is believed to be processed at the low-level of the visual cortex, such as V1. Adaptation to faces, on the other hand, can produce significant aftereffects in high-level traits such as identity, expression, and ethnicity. However, whether face adaptation necessitate awareness of face features is debatable. In the current study, we investigated whether facial expression aftereffects (FEAE) can be generated by partially visible faces. We first generated partially visible faces using the bubbles technique, in which the face was seen through randomly positioned circular apertures, and selected the bubbled faces for which the subjects were unable to identify happy or sad expressions. When the subjects adapted to static displays of these partial faces, no significant FEAE was found. However, when the subjects adapted to a dynamic video display of a series of different partial faces, a significant FEAE was observed. In both conditions, subjects could not identify facial expression in the individual adapting faces. These results suggest that our visual system is able to integrate unrecognizable partial faces over a short period of time and that the integrated percept affects our judgment on subsequently presented faces. We conclude that FEAE can be generated by partial face with little facial expression cues, implying that our cognitive system fills-in the missing parts during adaptation, or the subcortical structures are activated by the bubbled faces without conscious recognition of emotion during adaptation.

## Introduction

Because sensory systems exhibit short-term plasticity, prior experience can affect subsequent perception. Visual adaptation, a ubiquitous phenomenon of our visual system and a powerful psychophysical tool, provides a method to probe neural plasticity in vision. One consequence of visual adaptation is the visual aftereffect–the bias in our judgment of a visual stimulus presented after adaptation. Recent studies on the Facial Expression Aftereffect (FEAE) found that adaptation to a face with a particular expression can induce an opposite expression in a neutral face [1,2]. Xu et al. [3] showed that adapting to an image of a mouth or a simple curve can bias the judgment of facial expressions of subsequently presented faces, the FEAE. This suggested that the FEAE does not require the full face as an adaptor and is spatially localized, the latter further evidenced by the finding that the aftereffect only occurred when the adapting and test mouths were in the same retinal-image location. However, an important question remains. Do other parts of the face contribute to the FEAE, and if so, under what circumstances?

To address this question, we used the bubble technique [4] to manipulate the visibility of face features during adaptation. The bubble technique was originally developed to study the contribution of face features to face categorization tasks. By varying the position and size of small apertures, or “bubbles”, through which part of the stimulus is visible, one can identify/locate the most informative features in the stimulus [4]. For facial expression, placing bubbles near the mouth region makes emotion, e.g. happy or sad, more identifiable than placing the bubbles over other face regions [4,5]. In the current study, we used adaptors in which bubbles were placed either over the emotion-recognizable mouth regions or over the emotion-unrecognizable regions away from the mouth, in order to determine the contribution of different facial features to facial expression judgments and FEAEs. Specifically, we aimed to determine whether FEAEs occur following adaptation to static, emotion-unrecognizable bubbled-face images (experiment 1), whether FEAEs occur following adaptation to video-sequences of emotion-unrecognizable bubbled-faces (experiment 2), and whether FEAEs are affected by the size and location of the bubbled faces during adaptation (supplement experiment). The results of the study have enabled us to determine the extent to which the visual system is able to integrate information about facial expression that on its own is below the threshold for perceiving facial expression.

## Experiment 1

### Methods

#### Ethical Statement

All subjects were given written consent form prior to the experiment. The research has been approved by Internal Review Board (IRB), Nanyang Technological University, Singapore. The study was conducted according to the principles of the Declaration of Helsinki.

#### Subjects

A total of seven participants (five males and two females, average age: 26.7 years), with normal or corrected-to-normal vision, participated in the study. All participants were naïve to the purpose of the study. The study was approved by the Internal Review Board at Nanyang Technological University, Singapore.

#### Apparatus

Stimuli were presented on a 17-inch Samsung monitor (SyncMaster 793MB) with a refresh rate of 85 Hz and a spatial resolution of 1024 × 768. The monitor was controlled by an iMac Intel Core i3 computer. A chin rest was fixed at a distance of 75 cm from the monitor, making each pixel subtend 0.024°. Luminance was measured by a Minolta LS-110 photometer. Experiments were run in Matlab R2010a using Psychophysics Toolbox extensions [6,7].

#### Stimuli

The face images were obtained or derived from the Karolinska Directed Emotional Faces database (KDEF) [8]. All experiments were based on one person’s face selected from the dataset.

**Fig 1:**
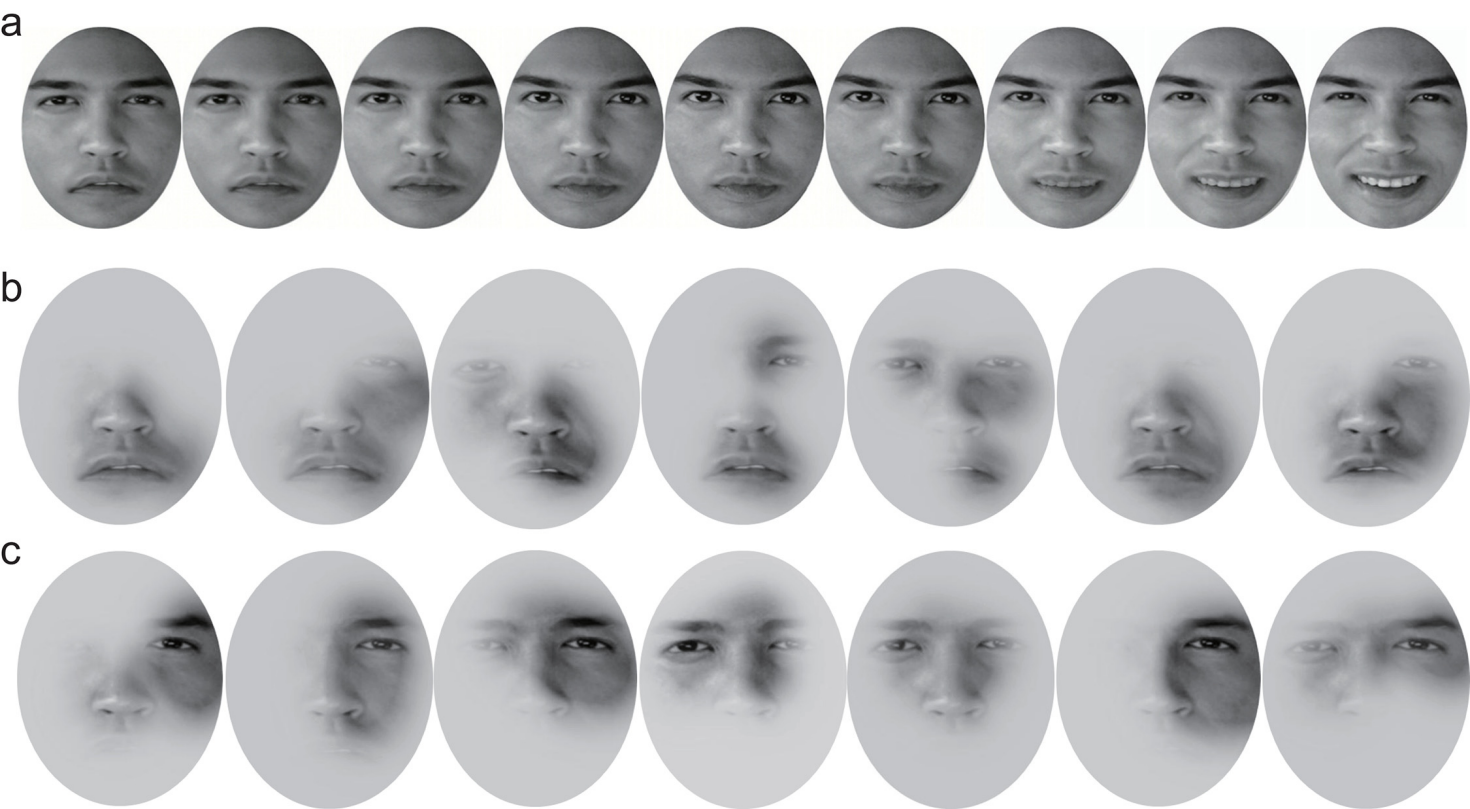
Demonstration stimuli for experiment 1. **a.** Test stimuli (varying from sad to happy). **b.** Examples of emotion-recognizable bubbled faces. **c.** Examples of emotion-unrecognizable bubbled faces. We have received informed consent (as indicated in the PLoS consent form) from the professional actor.

*Test stimuli*. Happy, sad, and neutral emotion face images of the same person were taken from the KDEF database. The faces were cropped to a size of 2.50° × 3.02° to ensure that they only included facial features. We used MorphMan 4.0 (STOIK Imaging, Moscow, Russia) to generate the intermediate facial expressions in steps of 0.05 by morphing the happy and neutral faces, and the neutral and sad faces. Twenty-one faces with equal-step changes were generated, with happiness ranging from 0 (sad) to 1 (happy) in proportions of 0.1, 0.2, 0.3, 0.4, 0.5, 0.6, 0.7, 0.8 and 0.9. Fig 1 showed demonstration stimuli derived from photographs of a professional actor (with written PLoS consent form as indicated).

*Adapting stimuli*. We developed four sets of adapting stimuli: the sad face and three sets of bubbled faces derived from the sad face. The three sets of bubbled face adaptors were: 1. mouth only; 2. emotion-unrecognizable static faces; and 3. emotion-recognizable static faces. To minimize adaptation aftereffects generated by local information, we enlarged the size of the adapting stimuli from 2.50° × 3.02° to 2.98° × 3.50°.

For the mouth only adaptor, we first added a grey mask onto the original photo, and then punctured the mask in the mouth region using a Gaussian aperture [4]. For the emotion-unrecognizable and recognizable static faces, we generated 180 adaptors by applying the bubble technique to the happy and sad faces (i.e. 90 images for each emotion). Specifically, four Gaussian apertures with a radius of 8 pixels and a fuzzy coefficient of 3 units (to blur the bubble surroundings, similar to the standard deviation of a Gaussian function) were randomly positioned on the two emotional faces. Next, we ran a pre-experiment (described in detail in the “*pre-experiment*” section in *Procedure*) to determine which of the bubbled faces were emotion-recognizable and which were emotion-unrecognizable. Based on the results of the pre-experiment, we then selected the emotion-recognizable faces as those judged > 94.4% in accuracy as emotion-recognizable, and the emotion-unrecognizable faces as those judged between 43.3% and 55% in accuracy as emotion-unrecognizable. We randomly selected nine faces from each category (see examples in Fig 1B and 1C). In the category of unrecognizable faces, the nine faces we selected were those with subtle emotional cues, such as eyes, but the information was too limited plus placed in the peripheral and therefore, people cannot tell the emotion. In this case, we have 36 faces in total (in each category: emotion × recognizability × number of faces = 2 × 2 × 9).

*Stimulus arrangement*. A black fixation cross (0.62 cd/m^2^ in Experiments 1 and 2, and 1.06 cd/m^2^ in S1 Text Supplementary Experiment) was presented on a white background (69.89 cd/m^2^ in Experiments 1 and 2, and 50.20 cd/m^2^ in S1 Text Supplementary Experiment) throughout the experiments. The fixation cross consisted of a vertical and a horizontal line subtending 0.34° and 0.048°, respectively. All visual stimuli were presented on the right side of the fixation cross at distances detailed below.

### Procedure

We used the constant stimuli and two-alternative forced-choice (2-AFC) research paradigm in all experiments.

*Pre-experiment (static face recognizability test)*. This experiment classified the static bubbled faces into emotion-recognizable and emotion-unrecognizable categories. Six subjects participated in this test. There were a total of 180 bubbled faces, half of which were happy faces and half sad faces. Participants were required to maintain their fixation on the cross throughout the experiment. Stimuli appeared for 4 s and participants judged the facial expression as happy or sad via a key press. If participants did not make a decision within 3 s after the stimulus disappeared, the face was classified as unrecognizable. All except 4 trials out of the total of 3240 produced responses within 3 s. From the results we identified 36 static faces divided into 4 categories: static recognizable happy; static recognizable sad; static unrecognizable happy; and static unrecognizable sad. The mean accuracy for static recognizable images was 98.2%, and for unrecognizable ones 51.03%. We also calculated response bias (c) ranging from -1 to 1, with positive values suggesting a conservative tendency (with “no” responses), negative values suggesting tendency of “yes” responses, and 0 suggesting no bias [9]. D-prime measures sensitivity, a score of 0 suggests inability to differentiate happy and sad [9]. In 2AFC task, a score of 0.75 is often used to define threshold, although other values may also be used [10]. The sensitivity of discriminating the sad unrecognizable static faces (the adapting faces) were: d′ = 0.23, and the bias *c* = 0.088. One-sample *t-*tests of the d′ revealed no difference from 0 (t_(5)_ = 2.38, P >. 05), indicate no discriminability in facial emotion for the sad unrecognizable static faces.

*Experiment 1*. This experiment determined whether the recognizability of the adapting facial expression affected the FEAE. The test stimulus was the full face taken from the face set (section *Test Stimuli*). There were five conditions: 1. full face adaptor (F); 2. mouth-only face adaptor (M); 3. static-recognizable face adaptor (SR); 4. static-unrecognizable face adaptor (SU); and, 5. no-adaptor baseline (N). For the static-recognizable and static-unrecognizable face adaptation conditions, one of the nine adapting faces from the corresponding group appeared as the adaptor. The adapting face varied from trial to trial in random order. The center-to-center distances from the fixation cross to the stimuli were 3.84° and 3.24° for the adaptor and test, respectively. This difference, together with the stimulus size difference (2.98° × 3.50° for the adaptor vs. 2.50° × 3.02° for the test), was designed to minimize the effects of localized, low-level contrast and luminance adaptation.

The five conditions were tested in a randomized block design, and the order of the test stimuli and adaptors was also randomized. Each condition was tested in two sessions. Within each session, the test stimuli were repeated 10 times. Each with-adaptation session typically lasted for 10 mins, and the baseline session took 3 mins. Participants had a ten-minute break between sessions to prevent carry-over effect (subjects may experience aftereffect after the adapting stimulus disappears) between two consecutive sessions.

**Fig 2:**
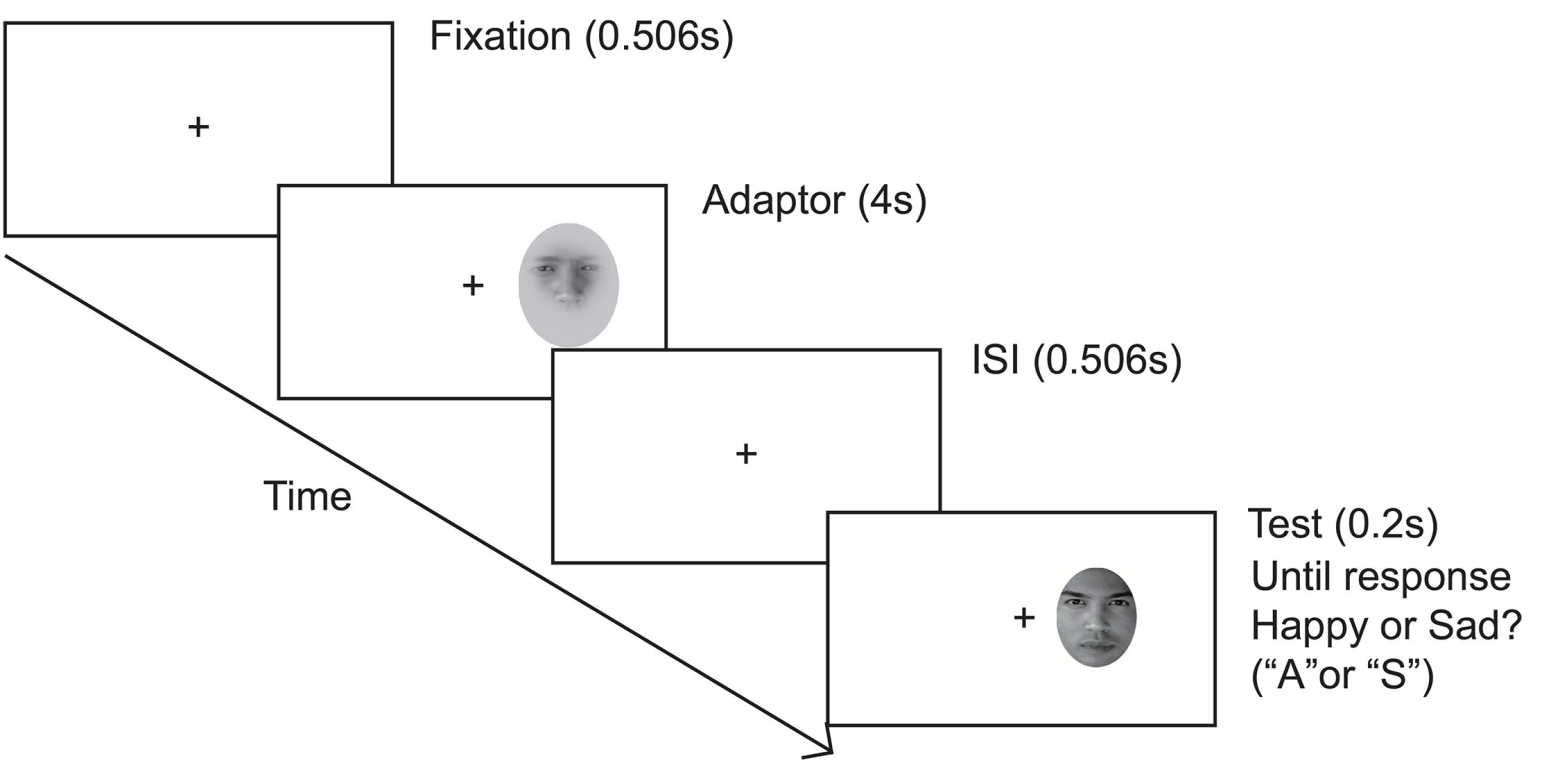
Trial sequence for the static emotion-unrecognizable adaptation condition in Experiment 1. Example is condition “SU”. Subjects fixated on the cross and pressed the space bar to initiate a trial block. After 506 ms, the adapting face appeared for 4 s. After a 506 ms inter-stimulus-interval (ISI), a test face appeared for 200 ms. A beep was played to remind subjects to press the appropriate key to report a happy or sad expression of the test face. In actual experiments, the fixation cross was always at the center of the screen.

Participants were required to maintain eye fixation on the central cross throughout the entire session. For the with-adaptation conditions, the adapting stimulus appeared and lasted for 4 s. After a 506 ms inter-stimulus-interval, the test stimulus was displayed for 200 ms, followed by a 50 ms beep to remind participants to respond (Fig 2). The screen remained blank until they responded. No feedback was given at any time.

*Data analysis*. Psychometric functions of the proportion of happy response trials as a function of stimuli proportion of happiness were fitted for each subject with the Sigmoidal (logistic) function in the form of *f*(*x*) = 1/[1+e^-a(x-b)^], where *b* is the test-stimulus parameter corresponding to 50% of the psychometric function [point of subjective equality (PSE)] and *a/4* is the slope of the function at the PSE. We used a two-tailed paired *t* test to compare subjects’ PSEs or the slopes for different conditions in the experiment. Aftereffect was measured as the difference between the PSE of an adaptation condition and the PSE of the corresponding baseline condition. We used the convention that repulsive aftereffects were negative. Analyses were performed in Matlab.

### Results

**Fig 3:**
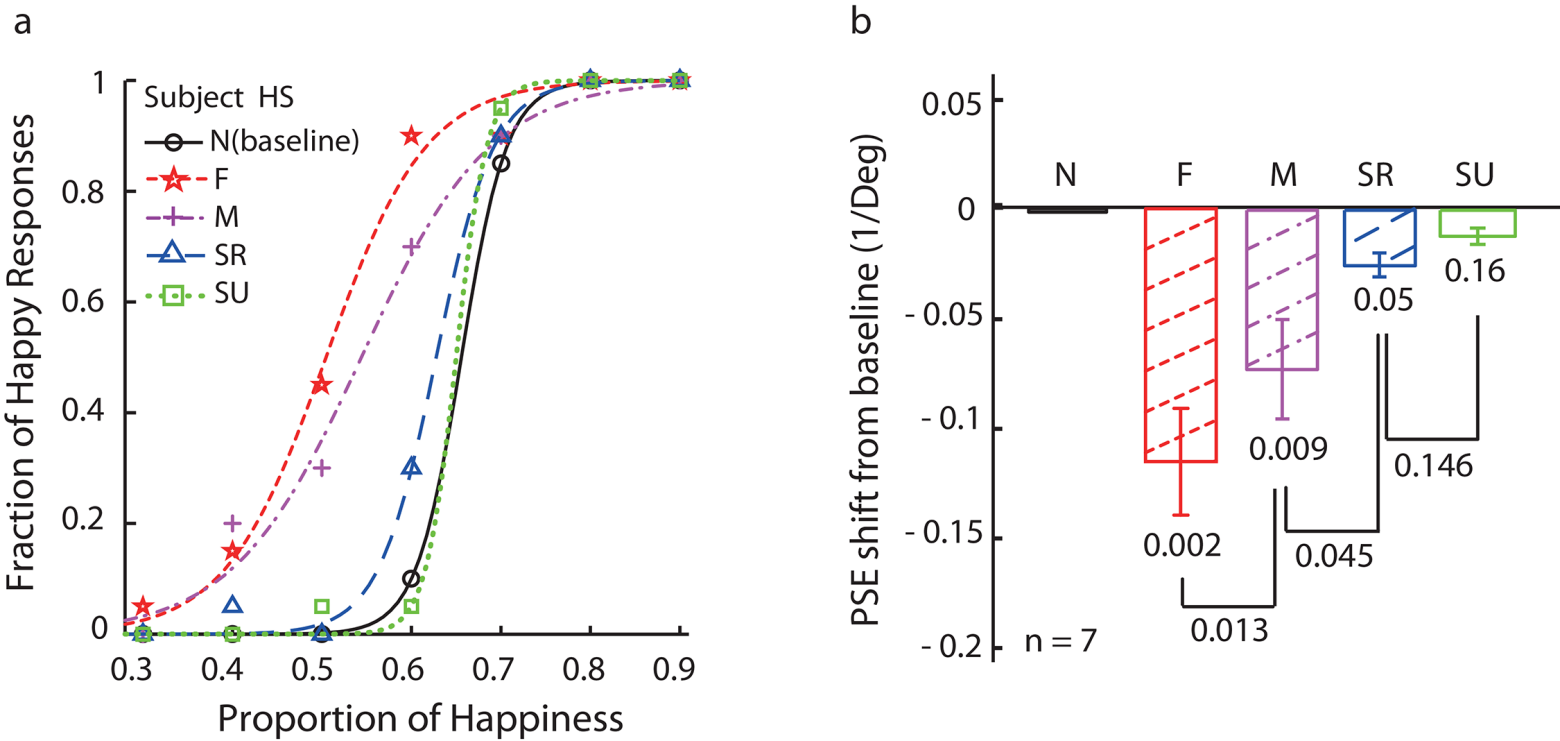
Facial expression aftereffect in Experiment 1. **a.** Psychometric functions from a naïve subject on facial emotion judgment (happy vs. sad). The five curves represent baseline condition (no adaptor, solid black circle, N), adaptation to a full sad face (dashed red star, F), adaptation to a sad mouth (dash-dot magenta cross, M), adaptation to a set of static recognizable faces (dashed blue triangle, SR), and adaptation to a set of static unrecognizable faces (dotted green square, SU). **b.** Summary of data from all seven participants. Error bars indicate SEMs and *p*-values were obtained by paired *t*-tests.

The results from one naïve subject are shown in Fig 3A. The figure plots the proportion of happy responses as a function of the stimuli’s proportion of happiness. The black curve is the baseline condition without adaptation (N). After adapting to the saddest face, the psychometric function for the full face adaptor shifted to the left–this is the expected FEAE [1, 2]. Adapting to the mouth-only image also produced a significant FEAE, as noted in our previous work [3]. However, new findings were observed for the bubbled face adaptors: the static emotion-recognizable adaptors produced a small facial expression aftereffect, whereas the static emotion-unrecognizable adaptors produced no aftereffect. Fig 3B shows the average FEAEs across the seven subjects for all four conditions. Each FEAE was calculated as the difference between the adaptation and baseline PSEs, and the error bars are standard errors of means (SEM) of the average FEAEs calculated across subjects. Two-tailed paired *t*-tests were conducted on the differences between the adaptation and baseline PSEs, and the resulting *p*-values are shown. Fig 3B shows that all FEAEs were significant, except the static emotion-unrecognizable FEAEs. We also compared the mouth and full face or emotion-recognizable FEAEs, and the differences were significant (*p* = .013 and *p* = .045, respectively). We then conducted a post hoc power analysis to see whether there is enough power to detect the difference. The powers for face, mouth, static recognizable and static unrecognizable face adaptation conditions (F, M, SR and SU) were 0.83, 0.62, 055 and 0.03, respectively, which were in line with their significance levels.

### Discussion

We measured the FEAE in full test faces using adaptors in which only parts of the face were visible at any one time. Adapting to a single face part, for which subjects were unable to recognize the emotion, did not generate a significant FEAE. In the static unrecognizable condition, the information was presumably too limited to activate the relevant perceptual mechanisms. Thus, participants were not able to consciously aware of the expression. How does this square with the results of studies that have manipulated awareness using methods other than bubbles? Three of these methods are crowding, continuous flash suppression (CFS) and masking.

First, the effect of crowding on adaptation. He et al. [11] and Rajimehr et al. [12] found that adapting to a crowded stimulus did not reduce an aftereffect in perceived orientation. However Blake et al. [13] showed that provided the crowded adaptor was low in contrast, a reduction in the orientation aftereffect caused by crowding could be obtained, suggesting that the high contrast adapting stimuli in the experiment by He et al. [11] and Rajimehr et al. [12] remained at saturation point even when crowded. For more complex features such as faces, adapting to a crowded face reduces the FEAE [14, 15]. Crowding mainly affects the ability to identify the details of the target rather than its visibility or presence [16]. In the present study subjects could see the static emotion-unrecognizable face adaptors but could not identify their emotion, and we did not find significant FEAEs, in line with Xu et al. [14] who also used static faces as adaptors.

Second, masking on adaptation. Of most relevance here is the paradigm in which an aftereffect is measured using an adaptor whose visibility is reduced or eliminated by a masking stimulus but which nevertheless continues to produce an aftereffect. Sweeny et al. [17] measured a shape aftereffect using an adaptor shape whose visibility could be reduced by a mask in the same eye (termed monoptic masking) or obliterated by a mask in the other eye (termed dichoptic masking). The adaptor shape was an open- or closed-curved oval shape and subjects were instructed to adjust a test oval or circle to a particular shape, with the degree of adjustment indicating the size of the aftereffect. The mask consisted of scrambled lines. Sweeny et al. [17] found a double dissociation for the open and closed adaptor curves, with the dichoptic masking eliminating the closed- but not open-curve aftereffect, and the monoptic masking eliminating the open- but not closed-curve aftereffect. These results suggest that the closed-curve but not open-curved aftereffects depend on visual awareness of the adaptor. Sweeny et al.’s finding is pertinent because an open curve is similar to the curve of a mouth whereas a closed curve describes the outline contour of a face. This is arguably consistent with our finding that the FEAE, which is based on adaptation of features such as the curve of the mouth, does not depend on visual awareness of those adaptor features.

Third, continuous flash suppression (CFS) on adaptation. In this paradigm, subjects are typically presented with a static stimulus in one eye and a series of rapidly changing stimuli in the other eye, resulting in suppressed perception of the static stimulus [18]. Moradi et al. [19] showed that reducing the visibility of the adapting face decreased the face identity aftereffect (distinct from the face emotion aftereffect, i.e. FEAE), and when the adapting face was invisible, the identity aftereffect vanished. Therefore, for face identity aftereffects, awareness of the adapting face appears to be necessary. Previous studies on the facial expression aftereffect (FEAE) using continuous flash suppression have produced mixed results. Adams et al. [20] found that FEAEs survived the effects of CFS, suggesting that FEAEs are not dependent on awareness of the adapting face. On the other hand, Yang et al. [21] found in an initial experiment that FEAEs did not survive CFS, suggesting that FEAEs do depend on awareness of the adapting face. However Yang et al. went on to show that the reduction in FEAE caused by the CFS was probably caused by inattention. When subjects attended to the suppressed adapting stimulus, the FEAEs were enhanced. In our study the adaptor was the only stimulus on the computer screen so we can assume that the participants were attending to the adaptor. Thus the results of FEAE studies using CFS to suppress awareness of the adaptor emotion would appear to be consistent with results of the present study.

To summarize this section: the findings from the aforementioned studies using crowding, masking or CFS are mixed with respect to the need for awareness of the adaptor emotion for producing FEAEs, with most studies supporting the idea that awareness is not necessary, as we find here. It is worth emphasizing however that in all three of the above methods the adaptor stimulus was physically complete when presented, so any reduction or abolition of perception must have been caused by competition between images, or rather between their neural representations [22]. However with the bubble method the adaptor is physically incomplete when presented. Therefore the discrepancies between previous studies and ours in terms of whether or not awareness of the adaptor emotion is necessary to produce FEAEs might ultimately be a consequence of the difference in the nature of the visual input, though for what precise reason is unclear. Since our brain prioritizes dynamic stimuli over static ones, as revealed by CFS, we conducted experiment 2 by adapting the subjects to dynamic bubbled-face to examine FEAE.

## Experiment 2

### Methods

#### Subjects

The same 7 subjects from experiment 1 participated in this study, four of which participated in the control experiment.

#### Stimuli

*Test stimuli*. The same set of test faces as experiment 1.

*Adapting stimuli*. We categorized the bubbled faces into 4 emotion/recognizability categories (e.g., sad recognizable) in Experiment 1, with a total of 36 faces (differ in emotion and location of the bubbles). To create the dynamic adaptors in Experiment 2, nine faces from the same category (e.g. sad emotion-recognizable) were combined into a video sequence in a random order. Only the sad emotion was used as adaptor. Thus, we have the two adapting videos: 1. dynamic emotion-unrecognizable video (DU), with all nine faces selected from the sad emotion-unrecognizable group; and 2. dynamic emotion-recognizable video (DR), with all nine faces from the sad emotion-recognizable group.

### Procedure

*Dynamic face video recognizability test*. All 7 subjects participated in this test after they had completed experiment 2. The procedure for the dynamic recognition task was the same as for the static recognition task, except for the duration. In this task each facial frame was presented for 435 ms with no between-frame interval such that the whole sequence took 3.92 s (see S1 Video for examples). Table 1 summarizes the results. The mean accuracy of the unrecognizable sad dynamic faces was 50.71%, which is close to chance performance, whereas the mean accuracy of the ‘unrecognizable’ dynamic happy face videos was above chance at 72.86%. To fully capture the discriminability of emotional faces by the participants, we calculated the d-prime (d′) and response bias (c). Our results for the unrecognizable sad adaptors were: d′ = 0.627 and bias c = 0.295. One-sample *t-*tests of the d′ revealed a marginal significant difference from 0 (t _(6)_ = 2.3, P = .06), indicating participants are more sensitive in discriminating the two emotions in unrecognizable dynamic settings compared with static ones (d′ = 0.23). Taking response bias into account, participants may have a tendency to respond to the stimuli as happy.

**Table 1:** Mean correct recognizable and unrecognizable emotional faces.

	Dynamic happy videos	Dynamic sad videos
Recognizable	91.43%	87.14%
Unrecognizable	72.86%	50.71%

Experiment 2 examined the FEAEs generated by dynamic adaptors. The procedure was identical to Experiment 1, except that the adaptor was a dynamic sequence of bubbled faces instead of a static bubbled face. In both conditions, each of the nine facial frames was displayed sequentially for 435 ms in a random order, and thus the duration for a dynamic adaptor in each trial was 3.92 s. The sequence of faces varied from trial to trial. We compared the aftereffect from the dynamic adaptor with that of the static adaptor from the previous experiment.

*Control experiment*. Four of the above seven subjects also participated in the following control experiment. The dynamic face video adaptor was comprised of nine faces, and hence contained more facial expression information than the static face adaptation condition. To examine whether the aftereffect difference between the dynamic and static face adaptors was caused by this information difference, we performed a control experiment in which the adaptors were single composite faces constructed by combining bubbled faces from the dynamic condition. To construct such composite faces, we first recorded the locations of all the bubbles of the nine selected faces from each category, then superimposed the bubbles and finally punctured them through a gray-masked face. We did this for the sad-unrecognizable, sad-recognizable, happy-unrecognizable, and happy-recognizable faces. We then tested the ability of the subjects to discriminate the facial expressions of the above four composite faces. The procedure was identical to the pre-experiment, except the face presentation duration was 435 ms in task one and 3.92 s in task two. The 435 ms duration was the same as that of a single frame in the dynamic video, and the 3.92 s duration equaled that of the entire dynamic video. We then tested subject performance using the composite face with bubbles from all sad-unrecognizable faces as the adaptor, and the adaptation durations were either 435 ms or 3.92 s.

### Results

**Fig 4:**
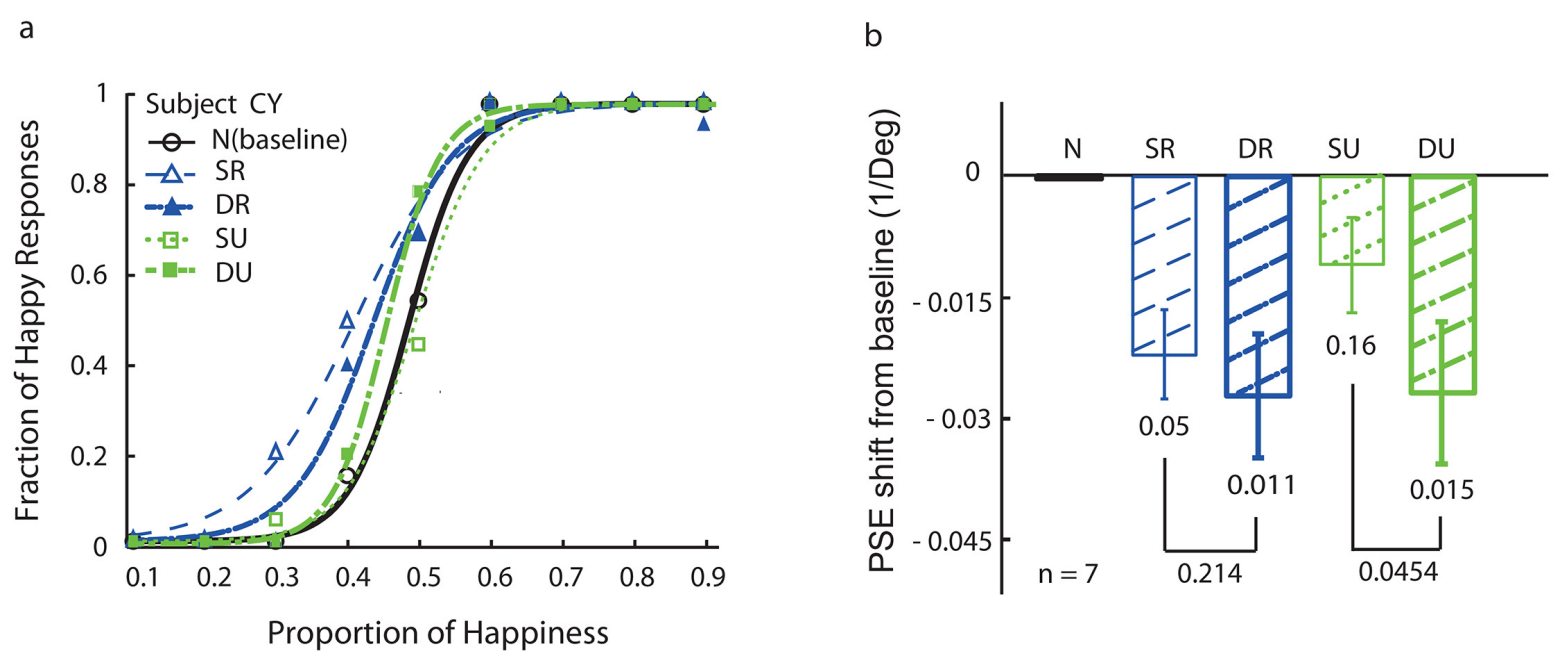
Facial expression aftereffect in Experiment 2. **a.** Psychometric functions from a naïve subject on facial emotion judgment (happy vs. sad). The five curves represent baseline condition (no adaptor, solid black circle, N), adaption to static emotion-recognizable face (blue dashed thin open triangle, SR), adaption to dynamic emotion-recognizable face video (blue dashed thick filled triangle, DR), adaption to static emotion-unrecognizable face (green dotted thin open square, SU), and adaption to dynamic emotion-unrecognizable face video (green dotted thick filled square, DU). **b.** Summary of data from all seven participants. The error bars indicates SEMs, and the *p*-values were obtained by paired *t*-tests.

In experiment 2, dynamic sequences of emotion-unrecognizable and emotion-recognizable bubbled faces were used as adaptors. Note that the adaptation duration was similar to the static face adaptation (3.92 s) and the data were collected from the same subjects in Experiment 1. Subject performance under these conditions was compared with the static face adaptation conditions (SR and SU) from Experiment 1 (Fig 4).

The results from one naïve subject (the same as in Fig 3A) are shown in Fig 4A. Interestingly, adapting to emotion-unrecognizable dynamic faces shifted the curve similarly to that of the other three adaptation conditions from the baseline condition. Fig 4B shows the mean FEAEs across seven subjects. Except for the static unrecognizable face adaptation condition (SU), all other adaptation conditions produced significant FEAEs. The powers for SR, DR, SU and DU were 0.55, 0.82, 0.03 and 0.85, respectively, in line with the significance of FEAEs.

**Fig 5:**
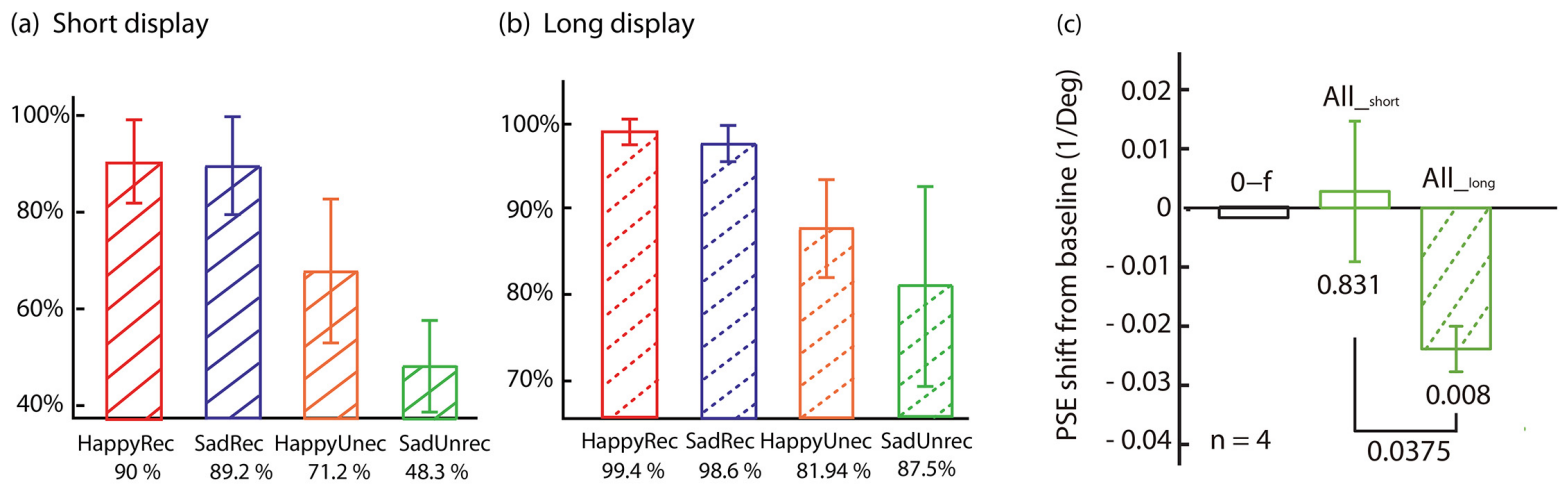
Control experiment. **a.** Judgment accuracy for short display (435 ms) of the single composite face for happy-recognizable (red bar) is 90.0%, 89.2% for sad-recognizable (blue bar), 71.2% for happy-unrecognizable (orange bar), and 48.3% for sad-unrecognizable (green bar). **b.** Judgment accuracy for long display (3.92 s) of the single composite face (99.4%, 98.6%, 87.5%, and 81.9% for happy-recognizable, sad-recognizable, happy-unrecognizable, and sad-unrecognizable, respectively). **c.** FEAEs from adapting to sad-unrecognizable faces at short adaptation duration (435 ms) and long adaptation duration (3.92 s), and baseline condition (0-f). The error bars indicates SEMs, and the *p*-values were obtained by paired *t*-tests.

*Control experiment*. Does the dynamic face video contain more information than the static face? We then tested the subjects’ ability to discriminate the composite faces, and also measured FEAEs using the composite faces as adaptors. In the discrimination task, subjects had to judge facial expression of the composite face. The face image was presented for 435 ms or for 3.92 s. Results revealed that discrimination depended on face stimulus duration (See Fig 5A and 5B). For the composite unrecognizable faces at short durations (435 ms, Fig 5A), accuracy was at chance level for sad-unrecognizable faces (48.3%), but above chance level for happy-unrecognizable faces (71.2%). For the composite recognizable faces accuracy was better: sad-recognizable = 89.2%; happy-recognizable = 90.0%. For long durations (3.92 s, Fig 5B), accuracy was much higher for all composite faces: happy-recognizable = 99.4%; sad-recognizable = 98.6%; happy-unrecognizable = 81.9%; and sad-unrecognizable = 87.5%. Therefore, longer display duration led to higher accuracy.

Adapting to the composite unrecognizable faces revealed that FEAEs were also duration dependent (Fig 5C). Adapting to sad-unrecognizable composites at short adaptation durations (435 ms) did not generate a significant FEAE (*p* = 0.831), whereas adapting to the same composite face at longer duration (3.92 s) did generate a significant FEAE (*p* = 0.008). Although it seems that the higher the classification accuracy in the adapting composite faces, the larger the facial expression aftereffect, the correlation between the two is not significant (*r* = -0.35, *p* = 0.395).

### Discussion

Although prolonged exposure to static emotion-unrecognizable face did not bias the emotion perception of subsequent faces (measured by FEAE), the dynamic counterpart did. As dynamic adaptors contained more information on facial expression than the single face adaptors, we performed a control experiment that collapsed all face parts from the dynamic sequence into a single face. When the composite face was presented for the same frame duration as the static adaptors (435 ms), it did not produce any significant FEAE. However, when presented for the 3.92s duration of the dynamic adaptor, the composite face did produce a significant FEAE.

Therefore, FEAEs from the dynamic visual adaptors most likely have resulted from the temporal integration of the sequence of briefly presented faces (435 ms per frame). We appear to be able to integrate the parts of faces into a whole across time. In our everyday visual experience of moving objects, we typically store information from previous images in a visual buffer and then combine them with incoming images [23–25]. Perceptual integration of this sort will generate a different pattern of brain activation compared with a static scene with the same (or even more) information. Using square apertures to reveal a background image, such as a face or building, James et al. [26] reported stronger and more sustained brain activation when viewing an aperture of changing images compared with a static scene.

Perceptual integration across time appears to be mediated by brain regions such as the right occipitotemporal and middle cingulate cortical areas [27, 28]. It has been shown that dynamic face videos activate the right posterior superior temporal sulcus (pSTS) and right anterior STS (aSTS), whereas static face images from these same face videos activate the right fusiform face area (FFA) and the right occipital face area (OFA) [29]. It is possible that the dynamic bubbled face videos used in our experiment not only activated face processing regions such as the STS, but also regions responsible for image integration, such as the right occipitotemporal cortex and middle cingulate cortex. Further investigations of the brain regions involved in dynamic bubbled face adaptation are needed.

## General Discussion

### Does attention facilitate adaptation to dynamic images?

When viewing a novel scene, people first distribute their attention across the scene before homing in on the object of interest [30, 31]. Focal spatial attention is then allocated to the target, facilitating categorization and additional processing [23]. Enhanced attention has been reported to amplify adaptation aftereffect in both low-level and high-level stimuli, such as curves and faces, respectively [32–35]. In our study, each frame of the dynamic face revealed very limited information about expression. To gain information on the entire dynamic video, subjects had to pay attention to the dynamic stimulus in sequence. Perception of dynamic images can induce greater and more sustained attention compared with static images. Therefore, it is possible that attention remained at a high level across the presentation of dynamic bubble faces. Our study supports the latter. This possibility invites future studies on eye movement patterns when viewing dynamic face videos.

In our experiments, since we did not monitor eye movements during face adaptation, subjects may have not always fixated on the point position and instead looked at the dynamic faces directly. This would have had two consequences: first it would have increased the accuracy for judging the static and dynamic faces during adaptation, and second it would have reduced the adaptation aftereffect which increases with adapting stimulus eccentricity [36]. These may be the reasons why we did not observe a strong correlation between the judgment accuracy of the adapting stimulus and the FEAE. However, we do not know whether subjects tended to break fixation more in the dynamic than the static faces, or if they did so equally to both. Using gaze-contingent crowding, a combination of crowding and eye movement tracking such that the crowded target emotional face changes to a neutral face at the moment when the subject breaks fixation, a novel method for unawareness by eye-tracking, Kouider et al. [37] found that crowded facial expressions biased evaluative judgments of neutral pictographs, and this bias works equally well for both static and dynamic faces. This suggests that strictly controlling the eye movement will have similar effect for static and dynamic face on evaluative judgment, but whether this affects its subsequent adaptation aftereffects to static and dynamic faces/videos requires further exploration.

### What are the possible neural pathways for perceiving bubbled faces and face emotion?

Using fMRI, Jiang and He [38] reported that when emotional faces were rendered invisible using binocular rivalry, activity in the Fusiform Face Area (FFA) was much reduced whereas activity in Superior Temporal Sulcus (STS) remained robust. The authors also found a high correlation between the neural activities in the amygdala and STS for invisible faces, but did not find a correlation between the amygdala and FFA. This is consistent with the functional and neural pathways previously established for face analysis by Haxby et al. [39]. Specifically, the changeable face properties such as facial expression appear to be mediated by STS, while the invariant face properties such as face identity appear to be mediated by the lateral fusiform gyrus. Haxby et al. further suggested that emotional information, especially when negative, is processed primarily by the amygdala, which is well known for processing fearful and sad information. Thus, for negative emotions, information may be processed through the subcortical regions (via superior colliculus, pulvinar to amygdala) which mediate input from both cortical and subcortical pathways [40, 41] and reflect a feed-forward pathway, i.e. without cortical feedback.

Affective stimuli could be detected by subcortical regions in a non-conscious, fast but coarse fashion ([40, 42] but [43]). When eye-whites from a fearful face was rendered invisible using backward masking, the amygdala still responded to it even though participants were not aware of the expression [44]. Patients with cortical blindness, which refers to vision loss due to damage of striate cortex, could nevertheless recognize/differentiate facial or body emotional information without consciously perceiving them [45, 46]. When stimuli were presented at the blind visual field, bilateral subcortical regions and the right FFA were activated [45, 46]. Single unit recordings in monkeys and clinical patients reinforced the involvement of subcortical areas in rapid face and facial emotion processing [41, 47, and 48]. Nguyen and colleagues [47] found that superior colliculus could respond to face-like patterns 25 ms after stimulus onset. Maior et al. [41] also reported that some pulvinar neurons could differentially responded to emotions in a short latency (<100 ms). Testing on neurosurgical patients with implanted depth electrodes, half of the tested amygdalae neurons responded to faces or parts of faces. About 10% responded differently to emotions, gender or identity. For the face-selective neurons, about 1/3 responded to bubbled faces, about 1/4 to whole faces, about 1/7 to eye cutouts and 1/5 to mouth (all in comparison to a face-scrambled baseline) [48]. It is thus reasonable to assume that our bubbled faces activated subcortical regions, which did not support conscious awareness of the emotion recognition but could detect salient facial features and contribute to the FEAE. Furthermore, the subcortical regions, such as superior colliculus, could send information to the pulvinar which further projects to the extrastriate cortex and induces FEAE [49, 50].

In addition, viewing the incomplete bubbled faces may activate the amodal completion process. Amodal completion refers to the situation when we perceive objects behind occluders, instead of seeing fragments; we view them as a whole. In a study conducted by Lin and He [51], they found that prolonged exposure to leftward/rightward moving diamond displayed behind circular apertures would bias the perception of a subsequent stimulus to the other end. Particularly, the adaptation aftereffect survived even when there was no overlap between the adapting and test stimuli. fMRI studies examined the neural mechanism of amodal completion, and found that the lateral occipital complex and dorsal loci were referentially responsive to occluded objects [52, 53].

For the limitation of the current study, future studies can be further explored. Firstly, we did not use an eye tracker to monitor eye movements to ensure participants fixate on the central cross throughout the whole experiment, however, given the fact that participants can learn the rules within a few trials; it should not be a big issue. However, with the eye tracking data, it would be interesting to see whether the significant adaptation aftereffect found in dynamic video condition can be, at least partially, attributed to the increased attention via microsaccade [54]. Given the moderate variation in the error bar, it is possible that participants may employ different amount of attention or strategies during the experiment. Secondly, the discrimination of the dynamic video was conducted afterwards, where a tendency of conservative judgment was observed. However, this result alone cannot nullify our findings, since there was no response bias in the initial discrimination test. The bias developed later may be due to long-lasting effect of adaptation [55]. Future studies may use emotions with different valence as adaptor to avoid the bias. We may also manipulate the consistency of emotional adaptor by changing its proportion of expression to see how it influences the FEAE. In addition, it would be interesting to test the temporal integration of emotional information to see whether it stands the current computation model using low-level stimuli [56].

## Supporting Information

S1 Dataset (ZIP)

S1 Fig Transfer of FEAE across size and location with dynamic face adaptation. **a.** Average FEAEs from all seven participants, calculated as mean PSE shift from baseline (N, black). The first three bars are for larger-, same-, and smaller-sized adaptors compared to tests (magenta–S_large_, blue—S_same_, and green—S_small_ solid hatched bars). The next three bars are the same conditions but at a lower location (magenta–L_large_, blue—L_same_, and green—L_small_ dotted hatched bars). **b.** Size and location conditions, blue curve: adaptor and test at same location; green curve adaptor at lower location than test. The error bars indicates SEMs, and the *p*-values were obtained.
(EPS)

S1 Text Supplement Experiment. (DOCX)

S1 Video Dynamic bubbled face video used in experiment 2 and supplement experiment. S1.a is emotion-recognizable video; S1.b is emotion-unrecognizable video.
(RAR)
