# Supplementary material for: Facial Expression Aftereffect Revealed by Adaption to Emotion-Invisible Dynamic Bubbled Faces
Source: PLoS One. 2015 Dec 30;10(12):e0145877. doi: 10.1371/journal.pone.0145877 (PMC4703136; doi:10.1371/journal.pone.0145877)
Supplement: S1 Text — (DOCX) [file pone.0145877.s003.docx]

**Supplement Experiment**

**Methods**

*Subjects.* Four participants from experiment 1 and additional three were recruited. Thus a total of 7 participated in this study (mean age: 24.7 years).

*Test and adapting stimuli.* The same as experiment 2.

**Procedure**

In the above experiments 1 and 2, although the adapting and test faces were of different size and presented at slightly different locations, local adaptation effects might still contribute to FEAEs. Therefore, we measured FEAEs using adaptor faces more markedly different in size and location from the test faces than in the previous experiments.

We used a 3 × 2 (size × location) within-subject design. Adapting stimuli were emotion-unrecognizable dynamic faces. The size of the adapting face was either larger, the same or smaller than the test face (3.50° × 4.23°, 2.50° × 3.02°, and 1.49 °× 1.82°, respectively). The adaptor was either centrally aligned with the test face or beneath the fixation cross without spatial overlap with the test face. The location and size of the test face was held constant (3.24° from the fixation cross) throughout the experiment. The procedure was identical to that of Experiment 2. There were a total of seven conditions: large adaptor at the same location (S_large_), same-sized adaptor at the same location (S_same_), small adaptor at the same location (S_small_), large adaptor presented lower (L_large_), same adaptor presented lower (L_same_), small adaptor presented lower (L_small_), and baseline condition with no adaptor (N).

**Results**

We examined whether the FEAEs generated by dynamic emotion-unrecognizable faces transferred across size and location. For size, we used adapting faces that were larger, the same size or smaller than the test face. For location, the adapting face appeared either at the same location as or lower than the test face location. Because the total number of conditions was relatively large (n = 7), we only presented the average data across subjects.

Results are shown in Fig S1a. When adapting stimuli were presented at the same location as the test face, both larger and same-sized adaptors generated significant FEAEs (*p* = 0.02 and *p* = 0.0057, respectively), but the smaller adaptor did not (*p* = 0.287). Thus, the FEAE produced by the dynamic emotion-unrecognizable adaptors appeared to be dependent on the relative size of the adaptor and test faces. Power analysis for larger-, same-, and smaller-sized adapting conditions were 0.67, 0.94 and 0.14, respectively, at the same location; and were 0.54, 0.83 and 0.13, respectively, when the corresponding adaptors moved to lower location.

**S1 Fig. Transfer of FEAE across size and location with dynamic face adaptation.**

**a.** Average FEAEs from all seven participants, calculated as mean PSE shift from baseline (N, black). The first three bars are for larger-, same-, and smaller-sized adaptors compared to tests (magenta – S_large_, blue - S_same_, and green - S_small_ solid hatched bars). The next three bars are the same conditions but at a lower location (magenta – L_large_, blue - L_same_, and green - L_small_ dotted hatched bars). **b.** Size and location conditions, blue curve: adaptor and test at same location; green curve adaptor at lower location than test. The error bars indicates SEMs, and the *p*-values were obtained

We then examined location specificity. Using the same-sized adaptor and test, we positioned the dynamic adaptor below the fixation point such that there was no overlap between the adaptor and test. We also applied the same manipulation to the larger and smaller adaptors. The results from the seven subjects are shown in Fig S1.a. When adapting stimuli were presented at the below-fixation-point location, both larger and same-sized adaptors generated significant FEAEs (*p* = 0.045 and *p* = 0.011), but the smaller adaptor did not (*p* = 0.451). Comparing FEAEs from different locations, the relative location of the adaptor and test did not matter for FEAEs produced by dynamic emotion-unrecognizable adaptors. This differed from our previous findings using static face adaptors [3, 21].

Fig S1.b shows the effects of size and location on aftereffect. The blue curve is for dynamic adaptor and test faces at the same location; the green curve is for dynamic adaptor below the test face. The two curves are in parallel for the smaller and same-sized adaptor and test, but crossed when the adaptor is larger than the test. However, the interaction between size and location was not significant (*p* = .527).

To summarize the results of Experiment 3, FEAEs using dynamic unrecognizable adaptors depended on the relative size (small vs. same/large) but not the location of the adaptor and test.
